# Case Report: Novel Anchoring Technique and Surgical Nuances for Trigeminal Ganglion Stimulation in the Treatment of Post-Herpetic Trigeminal Neuropathic Facial Pain

**DOI:** 10.3389/fpain.2022.835471

**Published:** 2022-03-17

**Authors:** Kunal Gupta

**Affiliations:** ^1^Department of Neurosurgery, Indiana University, Indianapolis, IN, United States; ^2^Stark Neuroscience Research Institute, Indiana University, Indianapolis, IN, United States

**Keywords:** trigeminal neuralgia, neuromodulation, neuropathic pain, stimulation, surgical technique

## Abstract

**Introduction:**

Trigeminal ganglion stimulation is a neuromodulatory surgical procedure utilized to treat trigeminal neuropathic pain. This technique involves the placement of a stimulating electrode adjacent to the trigeminal ganglion and can be trialed before permanent implantation. Wider adoption by surgical practitioners is currently limited by complications such as lead migration from the trigeminal ganglion, which can result in loss of therapy and cannot be rectified without repeat surgery. We describe a novel surgical modification that successfully anchors the trigeminal ganglion electrode long-term.

**Objective:**

To describe a novel surgical technique for the anchoring of trigeminal ganglion stimulation electrodes and a case report of a patient with post-herpetic trigeminal neuropathic pain treated with this approach.

**Methods:**

An electrode was inserted percutaneously through the foramen ovale into Meckel's cave, adjacent to the trigeminal ganglion. The lead was anchored using a modification of an existing anchoring device, which was inserted into the buccal incision. The lead was connected to a generator for therapeutic stimulation. The location of the lead was followed radiographically using serial lateral skull radiographs.

**Results:**

A 74-year-old male with post-herpetic trigeminal neuropathic pain, who had failed prior surgical therapies, underwent trigeminal ganglion stimulation. The trial lead was anchored using standard techniques and migrated outward within 7 days, rendering the trial electrode ineffective. The permanent lead was anchored using the described novel technique and remained in position without clinically significant outward migration nor loss in targeted stimulation until the last follow-up at 6 months.

**Conclusion:**

Trigeminal ganglion stimulation is an effective therapeutic option for medically refractory trigeminal neuropathic pain. The novel surgical adaptation described prevents the outward migration of the lead and enables stable long-term lead placement.

## Introduction

Trigeminal neuropathy is a severe pain syndrome affecting the facial dermatomes innervated by the trigeminal nerve. It has been classified under a number of different classification systems (1, 2). Classical trigeminal neuralgia is typified by episodic lancinating facial pain in the dermatomes of the trigeminal nerve and is typically managed by surgical approaches such as radiosurgery (3), microvascular decompression (4, 5), and a percutaneous rhizotomy of the trigeminal ganglion (6–8). Trigeminal neuropathic pain syndromes, however, may respond poorly to traditional surgical approaches and neuromodulation is becoming a mainstay in management (5, 9–11). Trigeminal ganglion stimulation is a neuromodulatory therapy that can be utilized for the treatment of trigeminal neuropathic pain syndromes (12–15). This technique involves the placement of a stimulating electrode, typically a spinal cord stimulator electrode used off-label for this purpose, into the foramen ovale providing direct stimulation of the trigeminal ganglion and nerve. A recent large series described 59 patients treated with trigeminal ganglion stimulation with or without an additional peripheral trigeminal nerve branch stimulating electrodes (14). Successful trial (defined as >50% improvement in pain) was reported in 71% of the patients and permanent implantation was associated with a visual analog score (VAS) improvement of 2.49 points (14); 70% of the patients indicated for this surgery were diagnosed with painful trigeminal neuropathy and atypical facial pain. The programming parameters used to achieve these outcomes were also recently described (16). There were a number of complications observed in this large series, including erosion, infection, and lead migration, which potentially limit wider adoption of this surgical technique. Erosion can be tackled by the use of frameless navigation to aid needle placement in deeper tissues, and infection can be reduced by replacing the stimulation electrode between trial and permanent implantation (14). However, there are currently no techniques described for the prevention of lead migration. Here, we describe novel modifications to existing surgical techniques that prevent lead migration and anchor the permanent electrode at the site of insertion and provide an illustrative case vignette.

## Case Description

### Case Vignette

**Figure 1:**
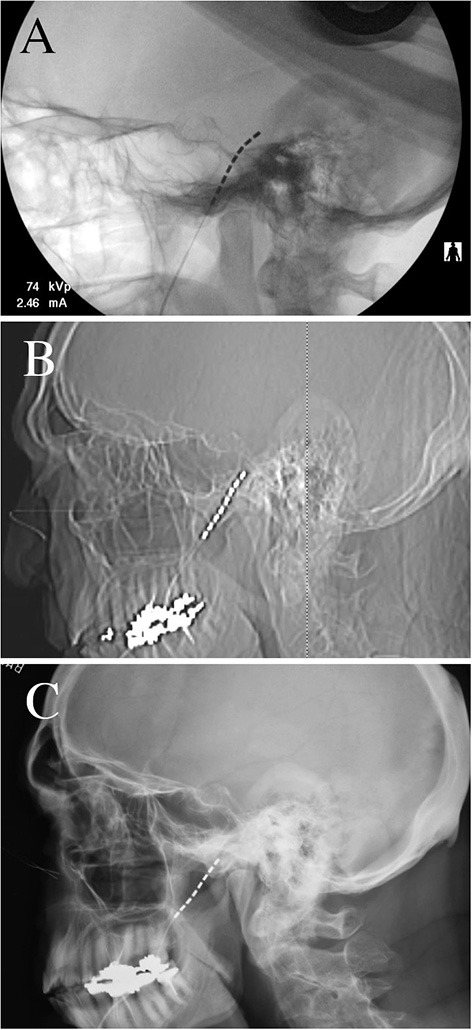
Radiographs of trial electrode placement. **(A)** Intra-operative lateral skull fluoroscopy image demonstrating placement of the electrode with the most distal contact within the foramen ovale. **(B)** Immediate postoperative lateral skull radiograph demonstrating that two-thirds of the electrode had migrated outwards (~35 mm) **(C)** 10-day postoperative lateral skull radiograph demonstrating complete migration of the trial electrode out of the foramen ovale, with no electrode contacts adjacent to the trigeminal ganglion.

**Figure 2:**
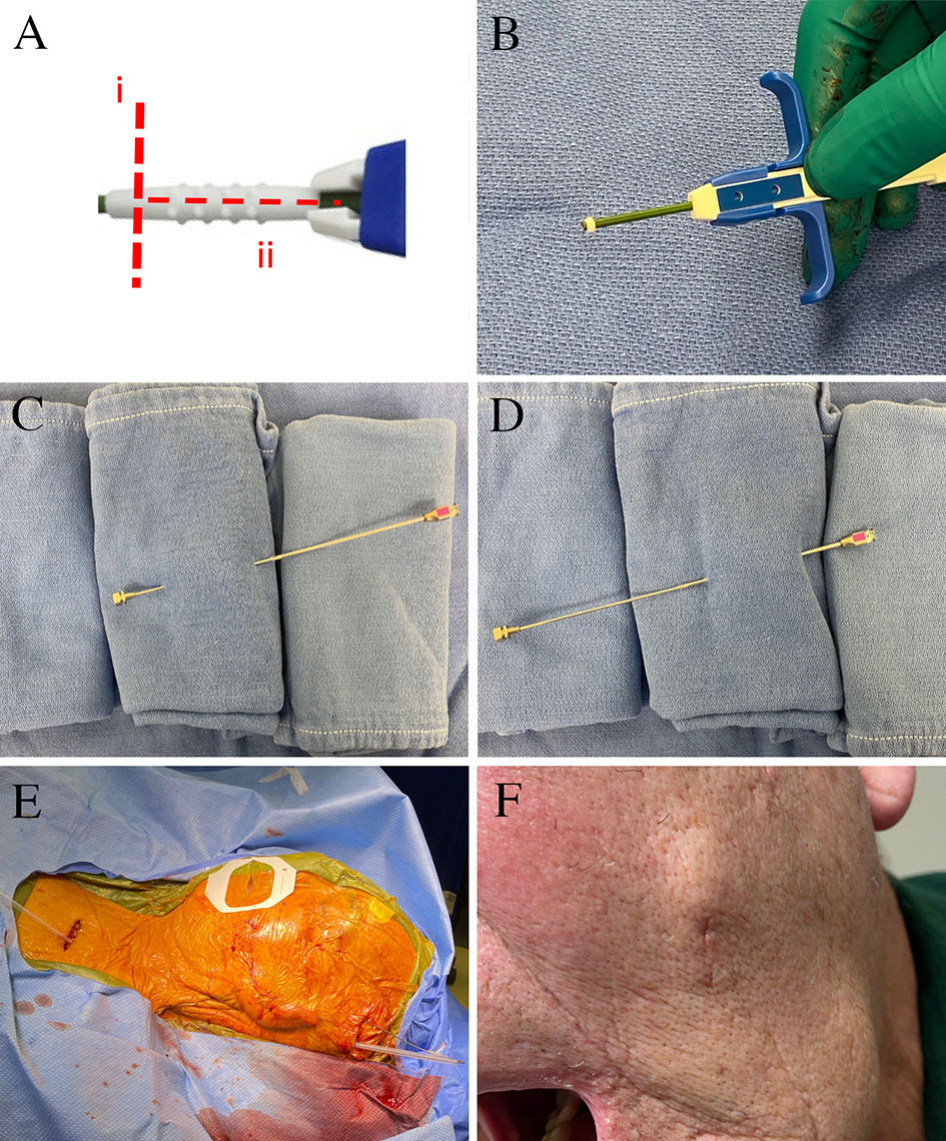
Surgical images for permanent electrode placement. **(A)** The manufacturer-supplied anchor is modified by (i) a circumferential cut to create a 2-mm ring at the end closest to deployment, and (ii) a longitudinal cut that allows the remaining anchor to be removed. **(B)** The injectable anchor is modified using a scalpel to leave a 2-mm ring at the tip. The remaining anchor is opened longitudinally and removed. This is implanted over the lead in a 3-mm buccal incision. The lead was tunneled from the buccal incision to the temporal region using a reverse-tunneled Tuohy needle: **(C)** the stylet is passed from the buccal incision to the temporal incision, **(D)** the hollow Tuohy needle is then inserted over the tip of the stylet at the temporal incision, and tunneled back to the buccal incision. **(E)** The lead was then tunneled over the pinna and to the subclavicular generator incision. **(F)** The left buccal incision healed well without significant cosmetic concern.

**Figure 3:**
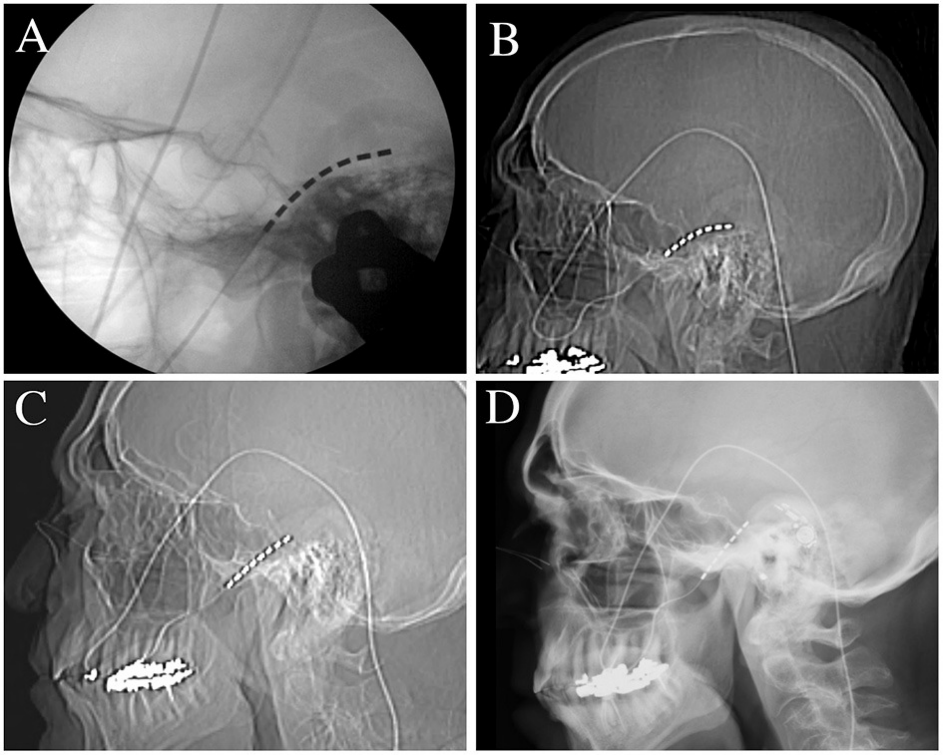
Radiographs of permanent electrode placement, secured with the novel anchoring technique. **(A)** Intra-operative lateral skull radiograph showing placement of the lead within the foramen ovale. **(B)** Immediate postoperative lateral skull radiograph showing preserved lead position. **(C)** Lateral skull radiograph was taken 3-days after permanent electrode implantation showing mild descent of the electrode by 3 contacts (~13 mm). **(D)** Lateral skull radiograph performed at 6 months showing no further movement of the lead.

The patient is a 76-year-old male with left-sided post-herpetic trigeminal neuropathic pain for 5 years. He described developing herpes zoster in the left mid and lower face and buccal mucosa and subsequently developed continuous burning pain as well as ipsilateral hearing loss. Prior to the local presentation, he sought care elsewhere and underwent stereotactic radiosurgery which did not improve his pain. He subsequently underwent microvascular decompression, also before local presentation, after which he states that his V2 pain improved. However, he remained with persistent neuropathic pain of the left V3 distribution. Upon examination, he had reduced sensation to pin-prick in the left V3 distribution. He elected to proceed with the trigeminal ganglion stimulation trial. During trial electrode placement, the lead was placed into Meckel's cave (Figure 1A). When compared to intra-operative imaging, the post-operative lead position immediately demonstrated partial migration of the lead after trial-electrode placement (Figure 1B). However, sufficient contacts remained adjacent to the trigeminal ganglion and he received programming per the described protocols (16). When re-evaluated 10 days after trial electrode placement, he reported 2 days of 50% pain relief before the resumption of the pain. Upon imaging of his lead, it was apparent that the trial lead had migrated completely out of the foramen ovale (this was not apparent at the cutaneous anchor, which remained in position), possibly explaining the loss of therapy after 2 days (Figure 1C). Given that he had 2-days of benefit, with the loss of benefit potentially explained by the migration of the lead, he elected to proceed with permanent implantation. Permanent implantation was performed with the addition of the modified anchor as described below (Figures 2A–F). Immediately after surgery, the imaging demonstrated the minimal settling of the lead with no further migration for 6 months after surgery (Figures 3A–D). The patient reported stimulation-induced paresthesia in the left V3 dermatome and 50% pain relief until the last follow-up.

### Timeline

Radiographic assessments were performed as described in Table 1. Imaging was performed during surgery, immediately postoperatively, and at out-patient clinical assessment: after 10–14 days during the trial, 1 month, and 6 months after permanent placement.

**Table 1:** Time-course of clinical and radiographic assessments.

**Timing of evaluation**	**Intra-operative**	**Immediately post-operative**	**Within 2-weeks of surgery**	**1 month after surgery**	**6 months after surgery**
Trial	Radiographic	Clinical and radiographic	Clinical and radiographic	N/A	N/A
Permanent	Radiographic	Clinical and radiographic	Clinical and radiographic	Clinical and radiographic	Clinical and radiographic

### Surgical Technique—Electrode Placement

Our operative technique has been well-described previously (14, 16). Trial electrode placement by conventional techniques remains prone to outward lead migration (Figures 1A–C). Here, our description focuses on technical adaptations for permanent electrode implantation, specifically, operative nuances aimed at complication avoidance and a description of the novel anchoring technique. Electrode placement was performed under general anesthesia. The patient's head was placed on a gel donut, and the Axiem frameless stereotactic system (Medtronic Inc, MN, USA) was used for navigation. The navigated stylet can be intermittently passed into the Tuohy needle for position checks during needle placement. The lack of rigid fixation of the head (by a radiolucent head holder for example) allows the head to be turned to tunnel the electrode to the subclavicular generator incision. Electrode placement is performed using a modification to Hartel's landmarks; a 14-gauge Tuohy needle is inserted into the cheek 2 cm lateral and 1 cm superior to the corner of the mouth. Typically, Hartel's entry point is 1 cm below the corner of the mouth (17), however, this area is hypermobile during the speech, eating, and mouth-opening, and may predispose to lead migration. Placing the entry point 1 cm above the corner of the mouth places the electrode insertion point in a less mobile area of the face and potentially reduces traction forces that may contribute to the descent of the electrode. The foramen ovale can be successfully cannulated with this modification, especially with the use of stereotaxy to guide needle placement. To avoid breaching the buccal mucosa, typically, a hand is inserted into the mouth to palpate the passage of the needle (and gloves changed on withdrawal from the mouth). However, since the buccal tissue thickens posteriorly, it can be challenging to track the needle, and the palpation method can predispose to more superficial needle placement. This may increase the chances of buccal erosion. The use of stereotactic navigation to guide the needle to the anterior border of the ramus of the mandible can help maintain the needle more centrally within the buccal tissue. To reduce the risk of infection from oral contents, especially for implantation of a permanent system, iodine-impregnated sterile surgical adhesive (e.g., Ioban) can be used to cover the orofacial apertures, and a small hole can be opened to allow placement of a finger into the mouth. Once the needle is navigated medially to and beyond the ramus of the mandible, the opening in the sterile drapes to the oral cavity can be covered with a sterile plastic adhesive (Figure 2F). The use of anticholinergic medications and requesting that the anesthesia provider perform suctioning of the oral cavity can also reduce saliva migration into the surgical field. The needle can then be navigated to the posteromedial portion of the foramen ovale. Stereotaxy can also be supplemented with intra-operative fluoroscopy. Once the foramen ovale is cannulated, the spinal cord stimulator guide-wire is bent to 30 degrees and guided posteromedially to create a path through the porus trigeminus along the cisternal segment of the trigeminal nerve, under continuous lateral fluoroscopy. The electrode (Vectris SureScan MRI 1x8 subcompact lead, model 977A160, Medtronic Inc MN USA) is then inserted through the Tuohy needle into the Meckel's cave. The use of this lead for trigeminal ganglion stimulation is currently off-label. The electrode is inserted until the most distal contact (contact 7, numbered 0–7) is within the foramen ovale and the most proximal contact (contact 0) is within the ambient cistern (Figures 1A, 3A–D). The Tuohy needle is removed under continuous lateral fluoroscopy to ensure that the electrode remains in place.

### Surgical Technique—Electrode Anchoring

For trials, a temporary anchor (Injex bi-wing anchor, Medtronic Inc MN USA) is deployed on the lead and secured to the skin using a non-resorbable suture for the duration of the trial. With this trial anchoring methodology, outward lead migration is expected (Figures 1A–C). We have specifically modified the permanent lead anchoring methodology to prevent lead migration and describe the technique here. Once the final position of the electrode is confirmed to be satisfactory, the permanent lead is anchored. To anchor the lead into position, an injectable spinal cord stimulator anchor is modified off-label (Figure 2A); the anchor is cut into a 2-mm ring using a scalpel at the end closest to the deployment of the anchor (Figure 2B). The remaining anchor is then cut longitudinally to allow its removal from the deployment stem. The anchoring ring is then deployed using the standard anchor deployment method onto the lead within the cheek skin incision at sufficient depth to allow skin closure. Tunneling through the cheek to the temporal incision can be performed using the same 14-gauge Tuohy needle; we advocate using a reverse tunneling method. The stylet is used alone to tunnel from the electrode incision to the temporal incision (Figure 2C), the hollow cannula is then placed over the tip of the stylet and reverse tunneled to the electrode incision (Figure 2D). This has the benefit of protecting the lead during tunneling and the thinner stylet may be less prone to unwanted penetrations of the buccal skin or mucosa. A further electrode anchor can be placed in the temporal incision and secured to the temporalis fascia if the lead is tunneled superior and posterior to the pinna to reach the generator pocket (Figure 2E). An alternative method is to tunnel directly from the cheek incision to the generator incision, though this is not within the author's current practice. The cheek incision was closed with a single subcuticular resorbable suture and healed well (Figure 2F). The lead was placed deep into the foramen ovale during surgery (Figure 3A) and remained in position immediately after surgery (Figure 3B). The lead descended 3 contacts (13 mm) by 3 days after surgery (Figure 3C) and remained in the same position 1 and 6 months after surgery (Figure 3D, 6-month image shown).

### Patient Perspective

The patient described that the shingles-related pain in his mouth was dramatically affecting his life; he has been unable to eat and drink, the pain interferes with his sleep and his wife is concerned that it is affecting their ability to “enjoy their retirement years.” He describes finding care to be difficult as many surgeons have stated that they are unable to treat his pain, and the interventions he has undergone have had limited efficacy. After undergoing trial electrode placement, he was cautiously optimistic, stating that the pain did not resolve but became tolerable with at least 50% improvement. He was very disappointed after the therapeutic effect of the trial was lost after 2 days, but reassured after we found that the lead had migrated. After permanent placement, he stated that he continued to have a 50% reduction in pain, able to eat and drink better, and participate more in daily life with his wife.

## Discussion

Trigeminal ganglion stimulation is a neuromodulatory therapy for the treatment of trigeminal neuropathic pain syndromes, which are typically less well-treated with conventional surgical approaches for classical trigeminal neuralgia such as microvascular decompression and percutaneous rhizotomy (though in this patient's case, he reported that his V2 facial pain improved after microvascular decompression). A number of alternative surgical approaches for trigeminal neuropathic pain have been described, including neuromodulation (e.g., trigeminal branch, trigeminal ganglion, and cervical spinal cord stimulation) and central ablative procedures (e.g., percutaneous tractotomy and caudalis dorsal root entry zone ablation). Each has challenges and benefits. Excellent results have been reported with peripheral nerve stimulation across a range of trigeminal pain disorders including classical trigeminal neuralgia, and trigeminal neuropathic pain syndromes secondary to multiple sclerosis, post-herpetic, and radiation-induced (18–22). However, to treat multiple dermatomes, multiple electrodes are required and, potentially additional generators, to accommodate multiple electrodes. Trigeminal ganglion stimulation is potentially attractive for its ability to target multiple dermatomes with a single electrode and can be combined with peripheral trigeminal branch stimulation (14, 16). This approach can help reduce the hardware burden. An approach we have utilized previously is an extensive trial with trigeminal ganglion and targeted trigeminal branch electrodes to identify the appropriate combination of leads for permanent implantation; limiting the required number of leads to 2 allows both leads to be inserted into a single dual-channel generator and reduce the patient's hardware burden. Furthermore, both trigeminal branch and trigeminal ganglion stimulation are associated with complications such as erosion (30%), infection (21–37%), and migration rates (11%) (13, 14). In our experience, the trigeminal branch stimulation of the V3 dermatome is particularly high risk for cutaneous erosion (likely due to the hypermobility of the lower jaw). Therefore, trigeminal ganglion stimulation, which also overlaps with the V3 distal nerve as it joins the ganglion, can be an effective substitute for the peripheral V3 lead and combined with single leads targeting either the V1 or V2 distribution. Interestingly, variable results have been reported for trigeminal branch and ganglion stimulation in the setting of post-herpetic trigeminal neuropathic pain, with some authors reporting no improvement and others reporting up to 70% of patients with at least 50% symptomatic improvement (13, 14, 20, 23). Given the limited surgical arsenal for this challenging pain disorder, the additional benefit of these neuromodulatory procedures is that they can be trialed prior to higher-risk central nervous system-ablative procedures.

There are numerous limitations to this description. This is an individual case report; larger series will be needed to validate the effectiveness of this anchoring technique. Outcome reporting is also short and longer follow-up periods will be needed to ensure that this anchoring methodology remains stable; however, no changes in lead position were seen between months 1 and 6. It is possible that the lead could be sutured within the buccal incision and this was not attempted due to the limited thickness of the buccal tissue and concerns about inflammation and erosion. The addition of a strain-relief loop at the incision may be effective, however, may be limited due to the thinness of the buccal tissue at the insertion point, potential risk of erosion, and adverse cosmesis of a larger incision. The described anchoring technique appears sufficient to anchor the lead, at least, over the described follow-up period in this single case report. Effectiveness, while not the focus of this report, can also be challenging to quantify due to the placebo effect, limitations of visual analog scores, and differences in reported effectiveness in various facial pain etiologies as described above.

In conclusion, a combination of neuromodulatory approaches is a valuable tool in the surgeon's armamentarium. Trigeminal ganglion stimulation is a nuanced surgical procedure, however, can be applied safely and effectively with attention to specific technical details.

## Data Availability Statement

The original contributions presented in the study are included in the article/supplementary material, further inquiries can be directed to the corresponding author.

## Ethics Statement

Ethical review and approval was not required for the study on human participants in accordance with the local legislation and institutional requirements. The patients/participants provided their written informed consent to participate in this study. Written informed consent was obtained from the individual(s) for the publication of any potentially identifiable images or data included in this article.

## Author Contributions

KG conceived of the case report, performed surgery, and wrote the manuscript.

## Conflict of Interest

The author declares that the research was conducted in the absence of any commercial or financial relationships that could be construed as a potential conflict of interest.

## Publisher's Note

All claims expressed in this article are solely those of the authors and do not necessarily represent those of their affiliated organizations, or those of the publisher, the editors and the reviewers. Any product that may be evaluated in this article, or claim that may be made by its manufacturer, is not guaranteed or endorsed by the publisher.
